# Protein SUMOylation regulates insulin secretion at multiple stages

**DOI:** 10.1038/s41598-019-39681-6

**Published:** 2019-02-27

**Authors:** Jeffrey S. Davey, Ruth E. Carmichael, Tim J. Craig

**Affiliations:** 1 Centre for Research in Biosciences, University of the West of England, Coldharbour Lane, Frenchay, Bristol, BS16 1QY UK; 2 College of Life and Environmental Sciences, Geoffrey Pope Building, University of Exeter, Stocker Road, Exeter, EX4 4QD UK

## Abstract

Type-II Diabetes Mellitus (T2DM) is one of the fastest growing public health issues of modern times, consuming 12% of worldwide health budgets and affecting an estimated 400 million people. A key pathological trait associated with this disease is the failure of normal glucose-stimulated insulin secretion (GSIS) from pancreatic beta cells. Several lines of evidence suggest that vesicle trafficking events such as insulin secretion are regulated by the post-translational modification, SUMOylation, and indeed SUMOylation has been proposed to act as a ‘brake’ on insulin exocytosis. Here, we show that diabetic stimuli which inhibit GSIS are correlated with an increase in cellular protein SUMOylation, and that inhibition of deSUMOylation reduces GSIS. We demonstrate that manipulation of cellular protein SUMOylation levels, by overexpression of several different components of the SUMOylation pathway, have varied and complex effects on GSIS, indicating that SUMOylation regulates this process at multiple stages. We further demonstrate that inhibition of syntaxin1A SUMOylation, via a knockdown-rescue strategy, greatly enhances GSIS. Our data are therefore consistent with the model that SUMOylation acts as a brake on GSIS, and we have identified SUMOylation of syntaxin 1 A as a potential component of this brake. However, our data also demonstrate that the role of SUMOylation in GSIS is complex and may involve many substrates.

## Introduction

Secretion of insulin from pancreatic beta cells is a critical process for the regulation of blood glucose homeostasis. Under normal conditions, a rise in blood glucose levels will result in an increase in glycolytic flux in pancreatic beta cells, resulting in a rise in the intracellular ATP/ADP ratio. The rise in the ratio of these nucleotides causes closure of the ATP-sensitive potassium channel (K_ATP_), resulting in depolarisation of the cell membrane and opening of voltage-gated L-type Ca^2+^ channels. Subsequent rapid influx of Ca^2+^ into the cells triggers the binding and fusion of insulin-containing secretory vesicles with the plasma membrane, resulting in insulin exocytosis. This process depends on the Ca^2+^-sensing protein, synaptotagmin, and the soluble N-ethylmaleimide Sensitive Factor attachment protein receptors (SNARE proteins), which provide most of the mechanical force for membrane fusion (for an extensive review see Rorsman and Ashcroft^1^). In Type-II Diabetes Mellitus (T2DM), one of the pathological changes which occurs is a reduction in this glucose-stimulated insulin secretion (GSIS) from pancreatic beta cells^2^, which contributes to the failure of blood glucose homeostasis symptomatic of this disease. Several dietary factors, including saturated fatty acids, have been shown to inhibit GSIS, however the molecular mechanisms for this inhibition are not fully elucidated^3^.

Although this process is well studied and mostly understood, it is not totally clear how it is regulated at the post-translational level by modifications such as SUMOylation, either under normal or pathological conditions. SUMOylation involves the covalent attachment of the Small Ubiquitin-like Modifier (SUMO), a peptide of 97 amino acids, to the primary amine groups of lysine residues via an isopeptide bond^4^. This modification requires the E2 enzyme Ubc9 and occurs mostly at the consensus motif ψ-x-K-E/D, where ψ represents a large, hydrophobic residue. In recent years, it has become apparent that SUMOylation plays a critical role in the regulation of several vesicle trafficking events, including neurotransmitter receptor surface expression and insulin release from pancreatic beta cells^5–12^. SNARE proteins of the syntaxin family are essential to catalyse the fusion of vesicles with the plasma membrane in all of these processes^13^, and SUMOylation of syntaxin1A has been shown to regulate the synaptic vesicle cycle^10^. Intriguingly, there is also evidence that SUMOylation is altered in pathological conditions including Alzheimer’s Disease^14,15^ and T2DM^16^, therefore raising the possibility that aberrant regulation of vesicle trafficking by changes in SUMOylation may underlie some aspects of the pathologies of these diseases.

In light of this, and the intriguing studies from the MacDonald group^17–19^, we studied the effect of manipulating global protein SUMOylation, and SUMOylation of syntaxin1A in particular, on GSIS from the rat insulinoma cell line, INS-1E. We observed that palmitate-induced deficits in insulin secretion were associated with an increase in cellular protein SUMOylation, however GSIS itself was not associated with significant changes in global SUMOylation profiles. We demonstrated that manipulation of protein SUMOylation by a variety of different tools alters GSIS, but in a complex manner suggesting multiple points of regulation. Further, we show that specific inhibition of syntaxin1A SUMOylation increases GSIS. We conclude that SUMOylation of syntaxin1A acts as a ‘brake’ on GSIS, but that SUMOylation likely regulates GSIS in a complex manner at many points of the pathway.

## Results and Discussion

### Glucose stimulated insulin release in INS-1E cells has no significant effect on global cellular SUMOylation

**Figure 1:**
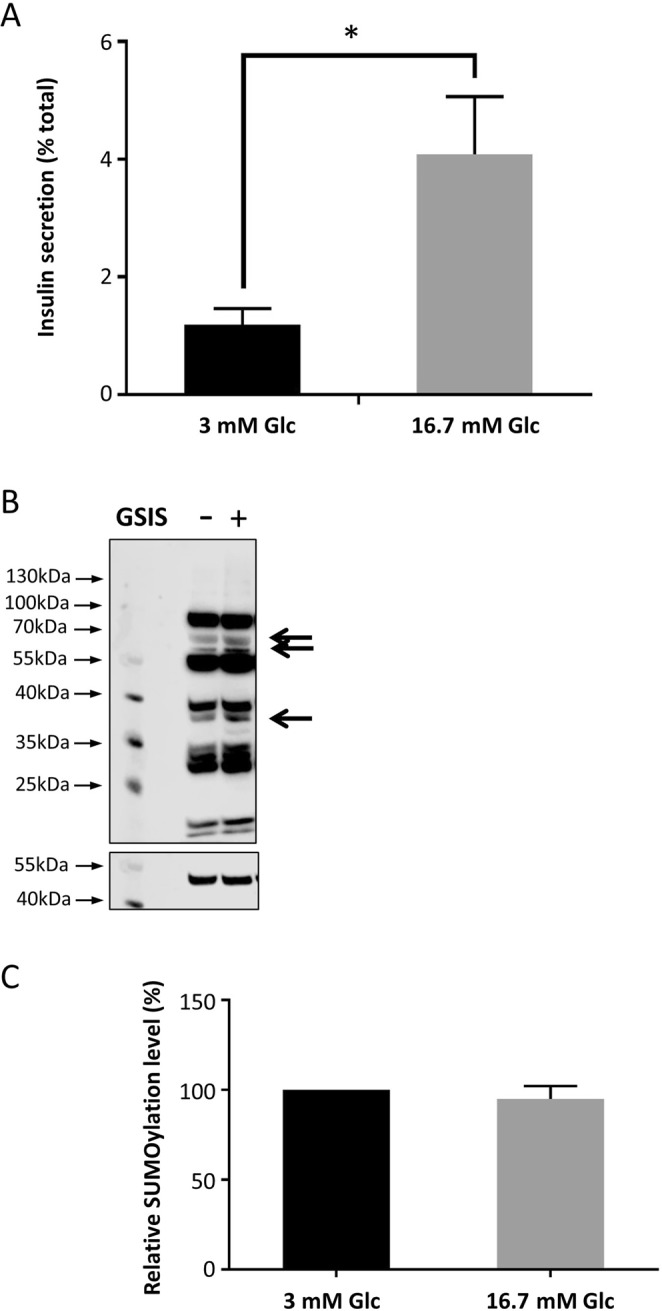
Glucose stimulation does not affect SUMOylation in INS-1E cells. (**A**) Comparison of % total insulin release by INS-1E cells in response to 3 mM and 16.7 mM glucose stimulation for 1 hour, as measured by insulin ELISA. *p < 0.05, Student’s t-test (n = 6). Note that subsequent insulin secretion results are presented as fold increase in secretion in the 16.7 mM glucose stimulated cells over the 3 mM glucose stimulated cells. (**B**) Representative Western blot for SUMO-1 (above) and alpha-tubulin (below) of whole cell lysates from cells treated with 3 mM glucose (**GSIS−**) or 16.7 mM glucose (**GSIS+**). Arrows indicate bands which potentially change on GSIS, suggesting that some substrates’ SUMOylation states are altered during GSIS. (**C**) Quantification of whole cells SUMOylation levels normalised to a-tubulin levels for cells treated with 3 mM and 16.7 mM glucose for 1 hour. Values are expressed as a % of 3 mM value for each replicate. Data are presented +/− SEM, n = 6.

Previous studies have reported a role for protein SUMOylation in the exocytosis of neurotransmitter-containing synaptic vesicles and insulin granules^10,17^. Additionally, various direct stimulations of neurones have been shown to result in changes in global cellular SUMOylation levels^11,20^. In order to investigate the response of SUMOylation in INS-1E cells to stimulation by glucose, we performed standard GSIS experiments in INS-1E cells (as detailed in Materials and Methods) and verified that our protocol resulted in robust insulin secretion (Fig. 1A). We then probed the lysates of the GSIS-treated cells with anti-SUMO1 antibodies (Fig. 1B). Densitometry analysis of the whole lanes revealed no significant changes in protein SUMOylation on glucose stimulation (Fig. 1C).

These results suggest that large-scale changes in cellular SUMOylation by SUMO1 do not occur during exposure of INS-1E cells to glucose concentrations sufficient to trigger robust insulin release. However, these data do not preclude a role for SUMOylation in this process as changes in the SUMOylation state of individual substrates (possible candidates for this are indicated with arrows) is often occluded in whole cell analyses such as these.

### Treatment of INS-1E cells with palmitate inhibits GSIS and increases SUMOylation

**Figure 2:**
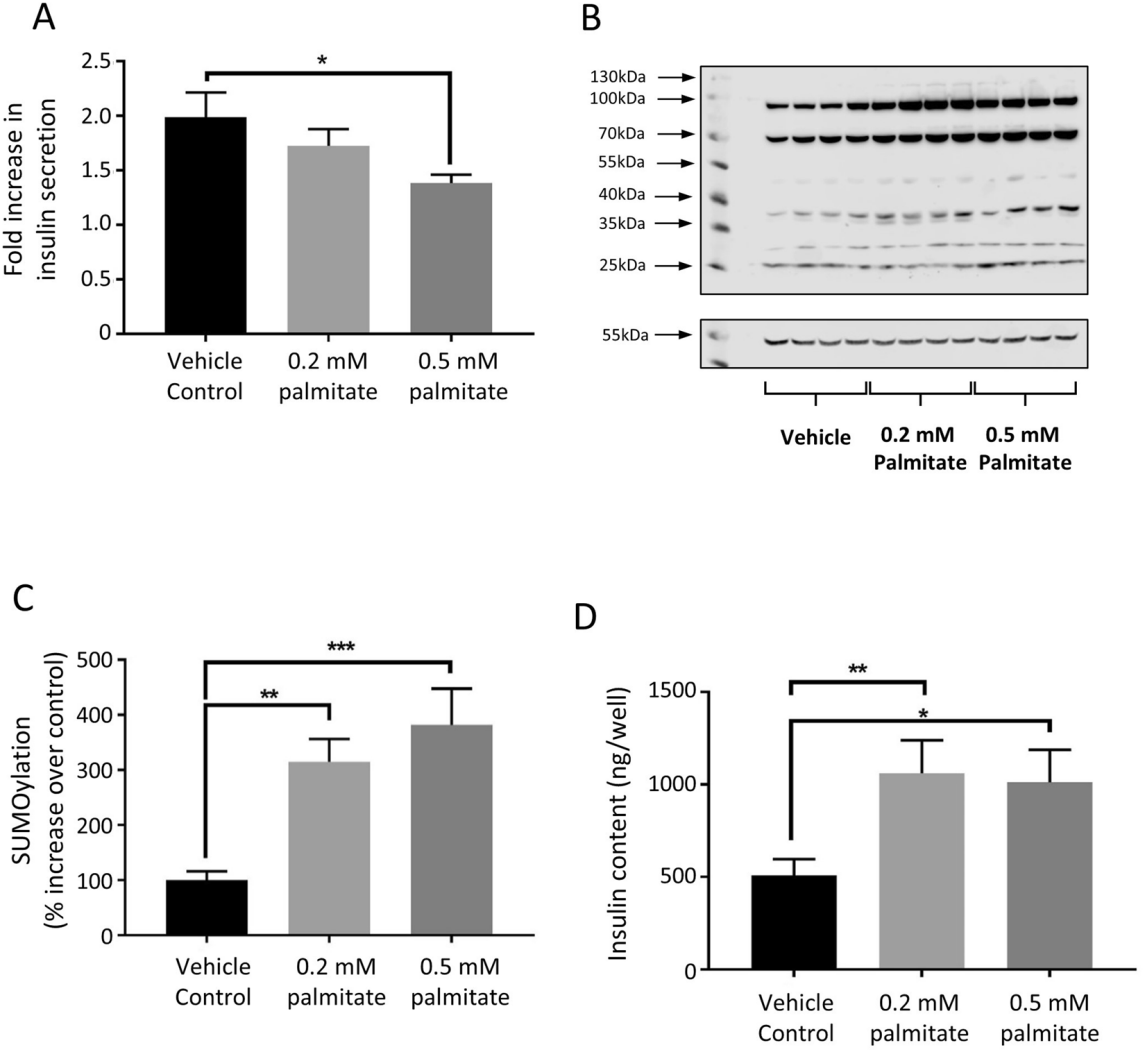
Palmitate treatment enhances SUMOylation and reduces insulin secretion. (**A**) GSIS expressed as fold increase in stimulation comparing 16.7 mM glucose stimulation to 3 mM glucose stimulation, in INS-1E cells exposed to 0.2 mM or 0.5 mM palmitate for 96 hours. *p < 0.05 in 1-way ANOVA (n = 6). (**B**) Representative Western blot showing total cellular SUMOylation levels (above) and alpha-tubulin (below) of cells treated as in (**A**). (**C**) Quantification of whole cells SUMOylation levels, normalised to alpha tubulin levels, in these cells. SUMOylation is expressed as % of that seen in vehicle control. **p < 0.01, ***p < 0.001, 1-way ANOVA (n = 16). (**D**) Total insulin content of cells, assayed by ELISA, exposed to vehicle control, 0.2 mM and 0.5 mM palmitate. *p < 0.05, ***p < 0.01, 1-way ANOVA (n = 6). Data are presented +/− SEM.

Changes in protein SUMOylation have been reported in different diseased states, including T2DM^3,16^. We therefore treated INS-1E cells with differing concentrations of palmitate, a free saturated fatty acid known to be a risk factor for T2DM^3^ and to inhibit GSIS^21,22^, in order to assess the effect of this treatment on whole cell SUMOylation. Our results indicate that 96 h treatment with 0.5 mM palmitate significantly decreases GSIS, as previously reported (Fig. 2A), and also significantly increases global SUMOylation by SUMO1 (Fig. 2B,C). Interestingly, in contrast to some previous reports, we also observed a significant increase in total cellular insulin content for cells treated with both 0.2 mM and 0.5 mM palmitate (Fig. 2D). The correlation of an increase in SUMOylation with a decrease in GSIS is in agreement with previously mentioned studies (e.g.^17,18^) suggesting that SUMOylation act as a ‘brake’ on insulin granule exocytosis. The effect of saturated fatty acids on beta cell function is clearly complex, however our data here imply that aberrant protein SUMOylation is one potential mechanism for saturated fatty acid-induced defects in insulin secretion. Interestingly, a previous study using high glucose treatment as a diabetogenic stimulus noted an increase in transcripts for SUMO paralogues and Ubc9^23^, and correlated this with an attenuation of the stimulatory effect of GLP-1 on GSIS. Therefore it is possible that SUMOylation changes are a general hallmark of a decline in beta cell health in response to metabolic challenge.

### Increases in cellular SUMOylation do not affect GSIS, but inhibition of deSUMOylation inhibits GSIS

**Figure 3:**
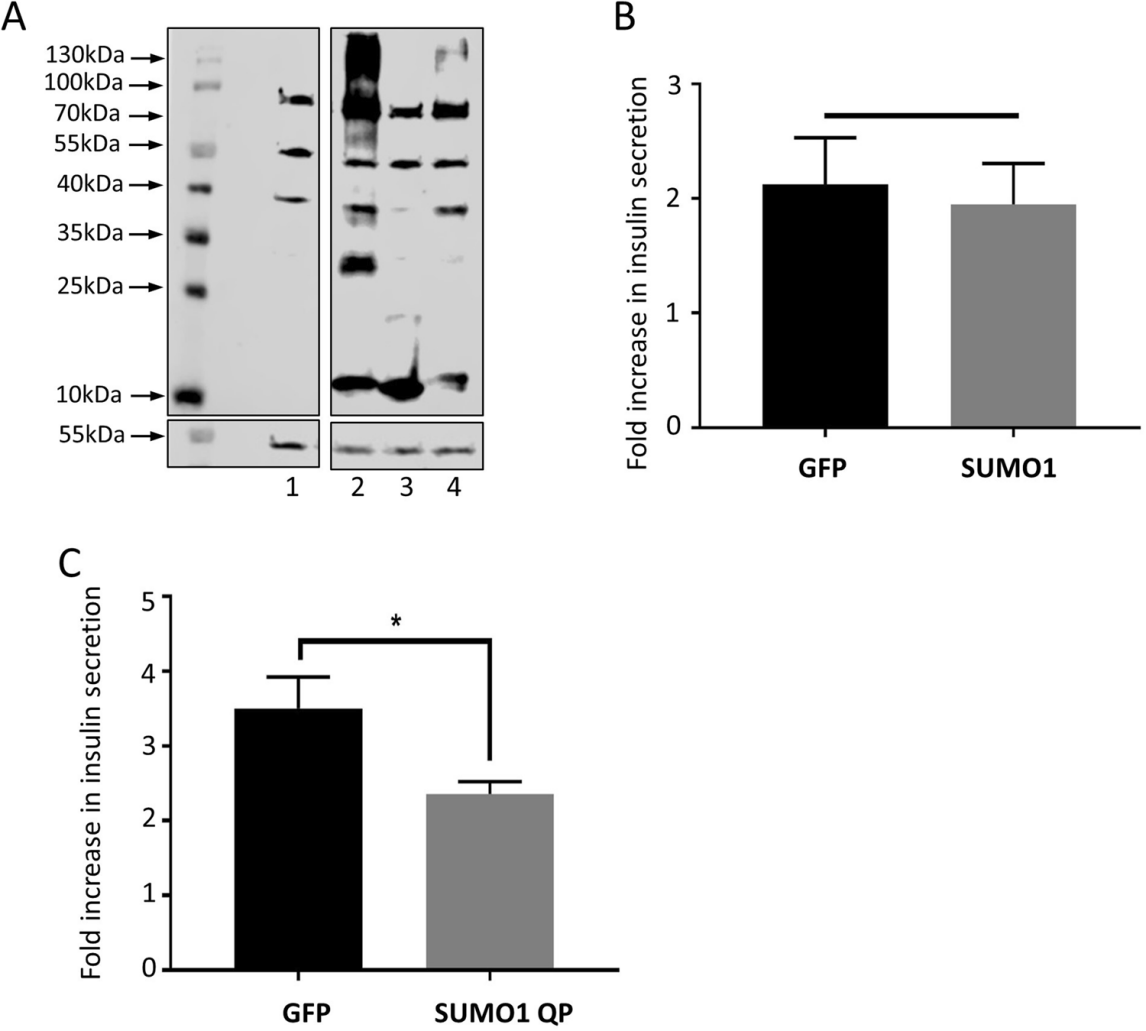
Inhibition of deSUMOylation inhibits insulin secretion. (**A**) Western blot showing whole cell SUMOylation (above) and alpha-tubulin (below) levels in cells infected with control virus (**1**), SUMO-1GG virus (**2**), SUMO-1dGG virus (**3**) and SUMO-1QP virus (**4**). The image shows lanes cropped from a single Western blot, to exclude an irrelevant lane that was run as part of the same experiment. For the full blot, please see Supplementary Information. (**B**) GSIS in INS-1E cells infected with control or SUMO-1GG overexpressing virus, expressed as fold increase in insulin secretion. No significant differences were noted (n = 6). (**C**) GSIS in INS-1E cells infected with control or SUMO-1QP overexpressing virus, expressed as fold increase in insulin secretion. *p < 0.05, Student’s t-test (n = 6). Full, uncropped blots are shown in Supplementary Data. Data are presented +/− SEM.

In order to further test whether SUMOylation acts to inhibit insulin granule exocytosis, we attempted to increase cellular SUMOylation by overexpression of mature SUMO1 peptides (SUMO1-GG) in INS-1E cells via Lentiviral transduction. We also overexpressed a mutant form of SUMO1 which is resistant to cleavage from substrates by SUMO-specific proteases (SUMO1-QP). As can be seen from Fig. 3A, overexpression of SUMO1-GG robustly increased total cellular SUMOylation, whereas a non-conjugatable mutant (SUMO1-ΔGG) did not. Overexpression of SUMO1-QP also increased SUMOylation levels, although not to the same extent as SUMO1-GG.

SUMO1-GG overexpressing cells showed identical levels of GSIS compared to GFP-expressing control cells (Fig. 3A), indicating that simply increasing cellular SUMOylation levels did not affect GSIS in INS1-E cells. However, a significant decrease in GSIS was observed in cells overexpressing SUMO1-QP (Fig. 3B). These data suggest that inhibition of deSUMOylation by SUMO1-QP attachment to cellular proteins inhibits GSIS, thus adding weight to the model that SUMOylation of one or more cellular components acts as a ‘brake’ on exocytosis, in broad agreement with previously mentioned studies and the data in Fig. 2.

### SENP1 overexpression inhibits GSIS in INS-1E cells

**Figure 4:**
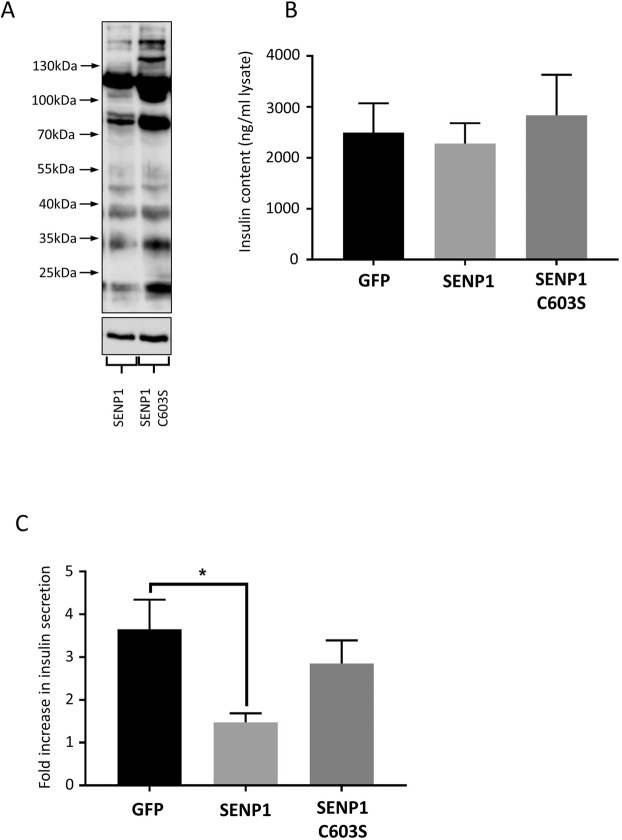
Perturbation of SUMOylation by overexpression of the SENP1 catalytic domain inhibits insulin secretion. (**A**) Western blots showing SUMOylation by SUMO1 in SENP1 + SENP1-C603S expressing INS-1E cells (above), and corresponding tubulin loading control (below). (**B**) Insulin content of INS-1E cells infected with the indicated lentivruses, measured by ELISA (n = 20). (**C**) GSIS in INS-1E cells infected with control, SENP1 or SENP1-C603S virus, expressed as fold increase in insulin secretion. *p < 0.05, 1-way ANOVA (n = 8). Data are presented +/− SEM. Full, uncropped blots are shown in Supplementary Data.

If it is correct that the major role of SUMOylation in GSIS is to inhibit insulin secretion, it follows that a reduction in global cellular SUMOylation levels should increase GSIS. In order to assess this, we used a Lentiviral transduction system to overexpress the catalytic domain of the SUMO-specific protease, SENP1, in INS-1E cells. Overexpression of SENP1, resulted in a noticeable reduction in SUMOylation by SUMO1, compared to the catalytically inactive mutant C603S (Fig. 4A). Unexpectedly, however, we found that overexpression of the catalytic domain of SENP1, but not the catalytically inactive C603S mutant, significantly decreased GSIS by approximately 50% compared to a GFP virus control (Fig. 4C).

The role of SENP1 in insulin secretion from beta cells is somewhat difficult to define, with several papers showing different effects. For example, studies by the MacDonald group have demonstrated that direct infusion of SENP1, during patch-clamping studies of insulin granule exocytosis, can increase depolarisation-induced exocytosis^17^, and that SENP1 is required for metabolism-related amplification in insulin granule exocytosis^18^, and normal insulin secretion in human islets^24^. However, other studies have demonstrated that SENP1 overexpression decreases GSIS^25^. Further, a recent study by another group has demonstrated that both ablation and overexpression of Ubc9 in mice resulted in lower levels of insulin secretion^26^. These results are not necessarily contradictory, instead we believe that they reflect the complex nature of the regulation of insulin secretion by SUMOylation, which likely has many roles at different stages of the pathway. Our data are in line with that obtained by Hajmrle *et al*.^25^, where the authors concluded that the SENP1-dependent decrease in GSIS was due to increased levels of apoptosis in SENP1 overexpressing cells. However, in our study there was no significant difference noted between total insulin content of cells infected with a GFP control virus and cells overexpressing SENP1 (Fig. 4B), arguing against increased cell death in our model. Interestingly, we noted a slight, statistically insignificant increase in insulin content in cells overexpressing the catalytically inactive SENP1 C603S. This result is possibly due to several outlying values in cells infected with this virus. A possible explanation for this discrepancy is that the previous study^25^ used full length SENP overexpression rather than the catalytic domain of SENP1. As the construct we employed is more indiscriminately localised than full length SENP1, it is possible that our data show the result of a more generalised reduction in cellular SUMOylation compared to previous studies. We also acknowledge, that due to differences in the viral transduction systems used in our study and that of Hajmrle *et al*., direct comparisons are difficult. These studies, therefore, combined with the results shown in Fig. 3, suggest that SUMOylation may play diverse regulatory roles at many stages in the insulin secretion pathway.

### Inhibition of Syntaxin1A SUMOylation enhances GSIS

We have previously demonstrated a role of syntaxin1A SUMOylation in the control of the synaptic vesicle cycle^10^. Given the striking similarities between synaptic vesicle exocytosis and insulin granule exocytosis, we investigated whether syntaxin1A SUMOylation also plays a role in GSIS. In order to investigate this, we utilised previously validated constructs^10^ which knock down syntaxin1A expression via shRNA and re-express either WT or a 3KR mutant of syntaxin1A, in which the three SUMOylatable lysine residues have been mutated to arginine (termed knockdown-rescue, or KD-rescue). This mutation has been demonstrated to inhibit SUMOylation of Stx1A whilst preserving its critical protein-protein interactions with other SNARE proteins^10^.

**Figure 5:**
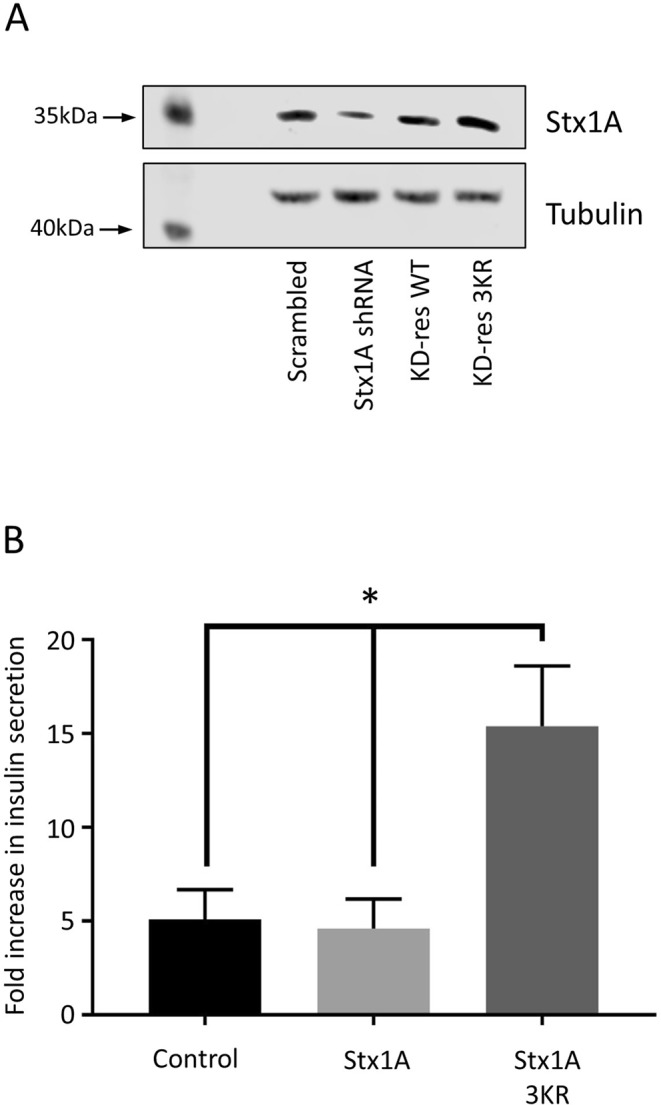
Inhibition of syntaxin1A SUMOylation enhances insulin secretion. (**A**) Western blot showing expression levels of syntaxin1A (above) and beta-tubulin (below) in cells infected with control, syntaxin1A shRNA, syntaxin1A knockdown-rescue wild-type (KD-res WT) or syntaxin1A knockdown-rescue 3KR (KD-res 3KR) virus. (**B**) GSIS in INS-1E cells infected with control, syntaxin1A knockdown-rescue wild-type (Stx1A) or syntaxin knockdown-rescue 3KR (Stx1A 3KR) virus, expressed as fold increase in insulin secretion. *p < 0.05, 1-way ANOVA (n = 5). Data are presented  + /− SEM. Full, uncropped blots are shown in Supplementary Data.

Strikingly, KD-rescue of syntaxin1A with the 3KR, non-SUMOylatable mutant, resulted in a statistically significant 3-fold increase in GSIS, compared to both the wild-type rescue and the GFP-expressing control cells (Fig. 5B). Importantly, as we found in rat primary neurones, our viral vectors do not result in an expression level of syntaxin1A beyond endogenous levels, indicating that this effect is not due to overexpression of the syntaxin 3KR mutant (Fig. 5A). These data therefore suggest that inhibition of syntaxin1A SUMOylation greatly enhances GSIS, in agreement with the proposed model whereby SUMOylation acts as a ‘brake’ on insulin granule exocytosis. These data are also in agreement with our previous findings demonstrating that SUMOylation of syntaxin1A inhibits its interaction with critical SNARE protein binding partners. However, it also suggests a fundamentally different role for syntaxin1A SUMOylation in neurotransmitter and insulin secretion, as inhibition of sytaxin1A SUMOylation was shown to decrease neurotransmitter release in our previous study^10^, in contrast to the increase in insulin release observed here.

## Conclusion

In this study, we have shown that protein SUMOylation in INS-1E cells is altered by exposure to the saturated fatty acid, palmitate, and that this is accompanied by a decrease in GSIS. These results, combined with our observations that inhibition of deSUMOylation inhibits GSIS and data from previous studies, adds weight to the model whereby protein SUMOylation plays an inhibitory role in the regulation of insulin granule exocytosis. Furthermore, our data imply that this mechanism may be altered by saturated fatty acids, a key nutrient risk factor in the development of T2DM, thus indicating that aberrant protein SUMOylation may play a role in the development of this disease.

What might the physiological role of this regulation be? MacDonald *et al*.^19^ postulate that SUMOylation of the syntaxin1A binding protein, tomosyn, acts as an inhibitor of insulin granule exocytosis which is relieved by increases in cellular glucose concentration, thus linking beta cell metabolism to SNARE complex formation. It is possible that syntaxin1A SUMOylation has the same role, however further investigation is required to confirm this. Insulin exocytosis must be tightly regulated, as aberrant insulin secretion can result in dangerous reductions in blood glucose levels; thus it is likely that the SUMOylation of these two proteins acts as a further regulatory ‘checkpoint’ to ensure that insulin release from pancreatic beta cells occurs in a precisely regulated manner in response to metabolic need.

It is important to note that, while our data are broadly consistent with previous studies, some of our results, e.g. the observation that both SENP1 overexpression and inhibition of deSUMOylation inhibit GSIS, imply a more complex role for SUMOylation in this process. This is perhaps unsurprising – there is no single unifying function for SUMOylation, much in the same way as phosphorylation, and it is entirely possible that multiple SUMO substrates have opposing functions in the GSIS pathway. Therefore, using instruments to globally increase or decrease SUMOylation are unlikely to give full mechanistic insight into the role of SUMOylation in these pathways. For this, manipulation of the SUMOylation of specific substrates is required (as we have attempted here for syntaxin1A). However, this requires identification of relevant SUMOylation substrates involved in the GSIS pathway, which remains technically challenging. This field, therefore, requires considerable further investigation before the exact roles of SUMOylation in beta cell function can be defined.

## Materials and Methods

### Reagents and Antibodies

All chemicals were purchased from Sigma-Aldrich unless otherwise stated. All tissue culture reagents were purchased from ThermoFisher. Mercodia Rat Insulin ELISA kits were purchased from Diagenics Ltd (Milton Keynes, UK). Anti SUMO1 (used 1:500), Ubc9 (used 1:1000) and syntaxin1A (used 1:250) antibodies were all purchased from Abcam Plc (Cambridge, UK). Anti beta-tubulin antibody (used 1:5000) was purchased from Sigma-Aldrich. Secondary antibodies were all purchased from Li-Cor Biosystems (used 1:10,000) (Cambridge, UK). INS-1E cells were a kind gift from Claes Wollheim (Lund, Sweden). HEK293T cells were a kind gift from Jeremy Henley (Bristol, UK), as were pXLG transfers vectors for lentivirus construction.

### Tissue culture

INS-1E cells were maintained in RPMI 1640 media supplemented with 5% FBS, L-glutamine, penicillin/streptomycin, 1 mM Na-pyruvate, 10 mM HEPES and 50 μM β-mercaptoethanol. For insulin ELISA assays, cells were plated at a density of 200,000 cells per well on a 12-well tissue culture plate and either infected with virus or treated with palmitic acid 24 h later. Insulin ELISA assays were performed 96 hours post-infection/treatment.

HEK293T cells were maintained in Dulbecco’s Modified Eagle Medium (DMEM, Sigma) supplemented with 10% FBS (Sigma) and L-glutamine. Serum-free DMEM was used in transfections for lentivirus production.

### Palmitic acid treatment

Palmitic acid was dissolved in 100% ethanol at a concentration of 100 mM, and then conjugated in a 5:1 molar ratio with fatty-acid free bovine serum albumin to make a stock solution of 10 mM. This was then mixed with INS-1E complete media to make the desired concentration of palmitic acid for cell treatment.

### Production of Lentiviruses

Lentiviruses were produced using the second generation pXLG vector^27^ in combination with the MISSION™ Lentiviral packaging system (Sigma-Aldrich). HEK293T cells were plated at a density of 2 × 10^6^ per 25 cm^2^ tissue culture flask. After 24 h, cells were transfected using 4 μg pXLG vector, 2 μg MISSION™ packaging mix and 24 μl PEI (1 mg/ml). After 48 h, media containing virus was harvested, centrifuged at 5,000 rpm for 10 min to remove dead cells, sterile filtered and frozen in aliquots at −80 °C. Test infections were performed to determine the volume of virus required for 100% infection of target cells (determined by fluorescence).

### Molecular biology

Validated constructs for syntaxin1A knockdown-rescue were used as previously described^28^, and cloned using standard molecular biology techniques. SUMO1-GG encoded the conjugatable human SUMO1 protein after SENP maturation, whereas SUMO-1ΔGG lacked the C-terminal diglycine motif required for conjugation^29^. To generate non-deconjugatable SUMO1-QP, glutamine residue 94 of SUMO1-GG was mutated to a proline, reducing the affinity of SUMO1 as a SENP substrate^30^. GFP-tagged human SENP1 catalytic domain, and the catalytically inactive C603S mutant, have been previously described^12^.

### Western blotting and quantification

For SDS-PAGE analysis, cells were lysed in lysis buffer (150 mM NaCl, 25 mM HEPES, 1% Triton X-100, 0.1% SDS, pH 7.4, Roche protease inhibitor cocktail) and loaded onto 12% polyacrylamide gels under denaturing conditions. Western blotting was performed using standard protocols. Visualisation was performed using a Li-Cor Odyssey Fc, and quantitation densitometry was performed using Li-Cor ImageStudio software.

### Insulin ELISA

Mercodia Insulin ELISA tests were performed according to manufacturer’s instructions. Insulin secretion assays were performed as follows: INS-1E cells were incubated overnight in complete media containing 3 mM glucose. This was replaced on the day of the assay with zero glucose Krebs-HEPES buffer (120 mM NaCl, 24 mM NaHCO_3_, 4.8 mM KCl, 2.5 mM CaCl_2_, 1.2 mM MgCl_2_, 0.1% BSA, 5.5 mM HEPES, pH 7.5). After 1 hour incubation at 37 °C, this was replaced with 0.5 ml KREBS-HEPES containing either 3 mM (control) or 16.7 mM (stimulated) glucose and incubated for a further hour at 37 °C. Buffer containing secreted insulin was removed and centrifuged to remove any cells. The remaining adherent cells were lysed in cell lysis buffer and used obtain total cellular insulin content levels. Typical dilutions used for the ELISA were 1:25 for secreted samples and 1:5000 for total insulin content. 10 μl of each diluted sample was used for the insulin ELISA. Insulin secretion was expressed as a percentage of total cellular insulin content, and from Fig. 2 onwards is expressed as fold increase in GSIS of 16.7 mM glucose compare to 3 mM glucose. The fold increase measure is used for most of this paper as it removes the variation in % insulin content release, which occurs in this cell line with passage number. Therefore, using % insulin release makes statistical comparison of different passage numbers and batches of cells more problematic.

Each experimental replicate is the mean of two duplicate wells for each condition. All values are expressed as mean +/− SEM.

### Statistical analysis

Student’s t-tests were used to compare 2 sets of values. For comparisons of more than 2 sets of values, 1-way ANOVA with Bonferroni’s post-hoc correction was performed. All statistical analysis was performed using GraphPad Prism software.

## Supplementary information

Supplementary Dataset 1

## Data Availability

All data generated or analysed during this study are included in this published article.
