# Supplementary material for: Protein SUMOylation regulates insulin secretion at multiple stages
Source: Sci Rep. 2019 Feb 27;9:2895. doi: 10.1038/s41598-019-39681-6 (PMC6393506; doi:10.1038/s41598-019-39681-6)
Supplement: Supplementary file 1 — Supplementary Dataset 1 [file 41598_2019_39681_MOESM1_ESM.pdf]

Protein SUMOylation regulates insulin secretion at multiple stages

Jeffrey S. Davey, Ruth E. Carmichael and Tim J. Craig*

Centre for Research in Biosciences, University of the West of England, Coldharbour Lane, Frenchay, Bristol, BS16 1QY, U.K.

*Please address correspondence to: tim.craig@uwe.ac.uk

Supplementary Information

Supplementary Figure 1 – Full Length Western blots

From figure 3C

SUMO1 blot

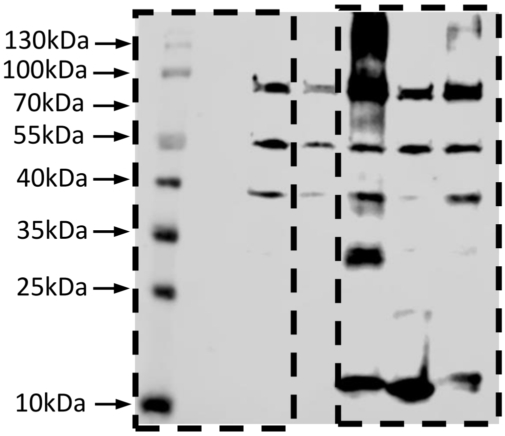

Tubulin blot

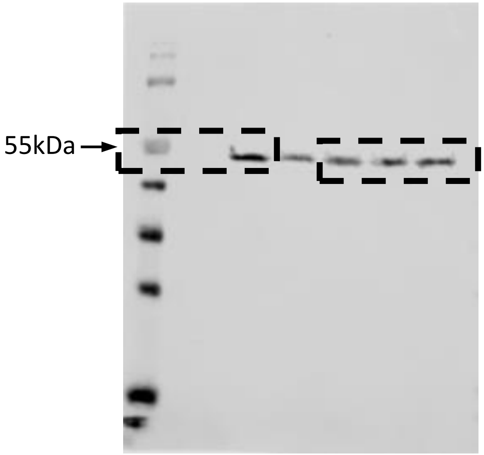

From figure 4A

SUMO1 blot

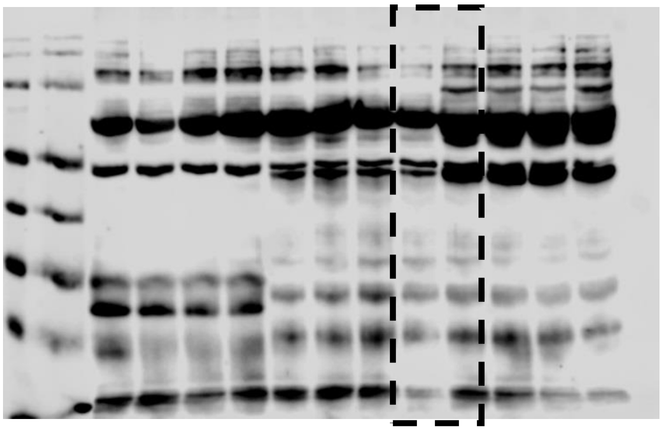

From figure 5A,B

Stx1A/tubulin blot

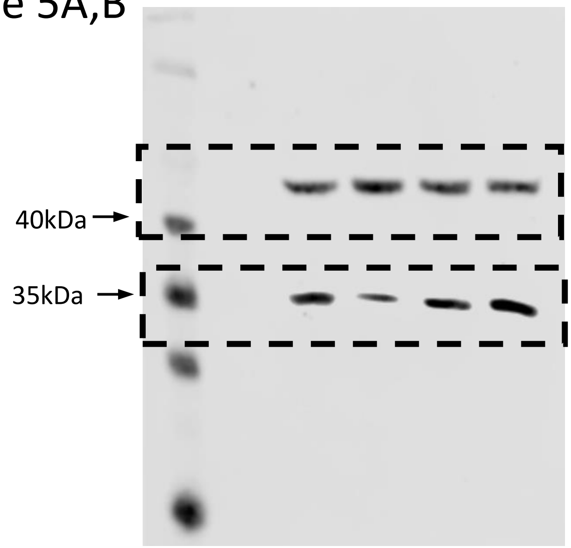

Supplementary Figure Legends

Supplementary Figure 1 - Full length blots

Full length, uncropped blots for all figures. Cropped areas shown in figures are indicated with dashed boxes.
